# Energy cost and lower leg muscle activities during erect bipedal locomotion under hyperoxia

**DOI:** 10.1186/s40101-018-0177-7

**Published:** 2018-06-19

**Authors:** Daijiro Abe, Yoshiyuki Fukuoka, Takafumi Maeda, Masahiro Horiuchi

**Affiliations:** 1 Center for Health and Sports Science, Kyushu Sangyo University, 2-3-1 Matsukadai, Higashi-ku, Fukuoka, 813-8503 Japan; 2 Faculty of Health and Sports Science, Doshisha University, 1-3 Miyakodani, Kyotanabe-shi, Kyoto, 610-0394 Japan; 3 Department of Human Science, Faculty of Design, Kyushu University, 4-9-1 Shiobaru, Minami-ku, Fukuoka, 815-8540 Japan; 4 Division of Human Environmental Science, Mt. Fuji Research Institute, 5597-1 Kamiyoshida, Fujiyoshida-shi, Yamanashi 403-0005 Japan

**Keywords:** Locomotion, Bipedalism, Optimal speed, Gait transition, EMG, Cost of transport

## Abstract

**Background:**

Energy cost of transport per unit distance (CoT) against speed shows U-shaped fashion in walking and linear fashion in running, indicating that there exists a specific walking speed minimizing the CoT, being defined as economical speed (ES). Another specific gait speed is the intersection speed between both fashions, being called energetically optimal transition speed (EOTS). We measured the ES, EOTS, and muscle activities during walking and running at the EOTS under hyperoxia (40% fraction of inspired oxygen) on the level and uphill gradients (+ 5%).

**Methods:**

Oxygen consumption $\left(\dot{V}{\mathrm{O}}_2\right)$ and carbon dioxide output $\left(\dot{V}{\mathrm{CO}}_2\right)$ were measured to calculate the CoT values at eight walking speeds (2.4–7.3 km h^−1^) and four running speeds (7.3–9.4 km h^− 1^) in 17 young males. Electromyography was recorded from *gastrocnemius medialis*, *gastrocnemius lateralis* (GL), and *tibialis anterior* (TA) to evaluate muscle activities. Mean power frequency (MPF) was obtained to compare motor unit recruitment patterns between walking and running.

**Results:**

$\dot{V}{\mathrm{O}}_2$, $\dot{V}{\mathrm{CO}}_2$, and CoT values were lower under hyperoxia than normoxia at faster walking speeds and any running speeds. A faster ES on the uphill gradient and slower EOTS on both gradients were observed under hyperoxia than normoxia. GL and TA activities became lower when switching from walking to running at the EOTS under both FiO_2_ conditions on both gradients, so did the MPF in the TA.

**Conclusions:**

ES and EOTS were influenced by reduced metabolic demands induced by hyperoxia. GL and TA activities in association with a lower shift of motor unit recruitment patterns in the TA would be related to the gait selection when walking or running at the EOTS.

**Trial registration:**

UMIN000017690 (R000020501). Registered May 26, 2015, before the first trial.

**Electronic supplementary material:**

The online version of this article (10.1186/s40101-018-0177-7) contains supplementary material, which is available to authorized users.

## Background

Erect bipedalism is an intrinsic gait pattern in humans. One of the most important biological benefits of the erect bipedalism has been reported to be economical for walking [1] and running [2]. There is a U-shaped relationship between the energy cost of transport per unit distance (CoT; J kg^−1^ km^−1^) and gait speed (*v*; km h^−1^) during walking as shown in Additional file 1: Figure S1. This means that there exists a specific walking speed minimizing the CoT. This specific walking speed is known as the economical speed (ES; km h^−1^) [3], which is close to preferred walking speed in healthy populations [4]. The most important factor for explaining the economical speed (ES) has been explained by the transfer efficiency between kinetic energy and gravitational potential energy [5, 6]; however, other factors, such as leg length [7, 8] and gravity [9], have also been considered. Our recent study showed an increase in the CoT values during walking under severe hypoxia (fraction of inspired oxygen; FiO_2_ (%) = 11%) compared to normoxia and moderate hypoxia at faster walking speeds [10]. This finding arose from a leftward (slower) shift of the U-shaped CoT-*v* relationship, resulting in a slower ES under severe hypoxia. In other words, the ES could be influenced by an alteration of the cardiorespiratory responses induced by different O_2_ conditions.

A linear relationship exists between the CoT and running speeds, meaning that there is an intersection between the U-shaped and linear CoT-*v* relationships, and this intersection is termed the “energetically optimal transition speed” (EOTS; km h^−1^) [11]. Since gait transition is triggered by the CoT [12, 13], the EOTS should relate to the gait transition. This concept has been widely accepted in discussions of bipedalism in other species [14, 15]. These results suggested that the CoT, which can strongly influence the ES and EOTS, may decrease by an acute hyperoxic exposure compared to a normoxic condition. Indeed, hyperoxia depresses peripheral chemoreceptor, resulting in a decrease in ventilatory response [16, 17]. As far as we know, the ES and/or EOTS have not been measured under different O_2_ conditions except for our recent hypoxic studies [10, 18]. Saving the whole-body energy expenditure at a given gait speed can provide an effective use of endurance capacity, that is, a possible combination of the rightward (faster) and downward (lesser metabolic) shifts of the U-shaped and linear CoT-*v* relationships will occur under hyperoxia relative to normoxia (Additional file 1: Figure S1). It is hypothesized that both ES and EOTS will be faster under hyperoxia than normoxia.

It has also been debated whether the EOTS is responsible for the natural gait transition [11, 19–22]. In association with the metabolic studies, several biomechanical studies reported that an abrupt increase in the *tibialis anterior* (TA) activity during walking can be another potential trigger for walk-run transition, although the TA mainly activates in the swing phase [23–26]. Considering these previous studies, gait selection would be dependent on both minimizations of the whole-body energy expenditure and muscular activity of the TA. We recently observed a decrease in muscle activity and lower shift of the mean power frequency (MPF; Hz) in the TA when switching from walking to running at the EOTS on the level and uphill gradients [18]. These results observed under normoxia (20.9% FiO_2_) and moderate hypoxia (15% FiO_2_) suggested that TA activity and its motor unit recruitment pattern were related to gait selection at the EOTS. *Gastrocnemius medialis* (GM) and *lateralis* (GL), antagonists of the TA, should also be examined, because these plantar flexors are substantially responsible for the forward acceleration [27].

In contrast to hypoxia, motor unit firing rates of the lower leg extremities increased under hyperoxia in comparison with normoxia [28]. It was also hypothesized that muscle activities and MPF would be higher under hyperoxia than normoxia during walking and running at the EOTS due to a presumably higher recruitment of glycolytic fibers. To test these hypotheses, our present study examined the effects of moderate hyperoxia (40% FiO_2_) on the ES and EOTS on the level and uphill gradients, because different gradient conditions would induce different leg muscle activation patterns. And then, muscle activity and motor unit recruitment patterns of the lower leg extremities were compared between walking and running at the EOTS under different FiO_2_ and gradient conditions.

## Methods

### Participants

The mean age, height, and body mass of 17 active male participants were 19.9 ± 0.9 years old, 1.69 ± 0.06 m, and 60.1 ± 7.9 kg, respectively (mean ± standard deviation; SD). In accordance with the Declaration of Helsinki, all participants were provided all information about the purpose and experimental protocols; a written informed consent was obtained from all participants. An ethical committee established in Kyushu Sangyo University approved all procedures of this study (H28-0001).

### Protocol

The FiO_2_ was set under normoxia (20.9% FiO_2_) and hyperoxia (40.0 ± 0.3% FiO_2_). The participants performed one of four cardiorespiratory measurements (2 FiO_2_ conditions × 2 gradients) once a day in random order. On each measurement day, they continuously walked and ran on a motor-driven treadmill (LABORDO LXE1200, Senoh, Japan) with a freely chosen step frequency at eight walking speeds (2.4, 3.1, 3.8, 4.5, 5.2, 5.9, 6.6, and 7.3 km h^−1^) on the level (± 0%) or uphill (+ 5%) gradients. Four running speeds (7.3, 8.0, 8.7, and 9.4 km h^−1^) were provided for running on both gradients, and 1 min standing rest was inserted among each running stage [18]. Four minutes were provided for each stage. These multiple gait speeds are enough to approximating reliable U-shaped and linear CoT-*v* relationships, contributing to a reliable evaluation of the individual ES and EOTS. One of the participants could not accomplish walking at 7.3 km h^−1^ on the uphill gradient and running at more than 8.0 km h^−1^, so that 6.9 km h^− 1^ were evaluated for uphill walking instead of 7.3 km h^−1^. For uphill running of this participant, 6.6, 6.9, and 7.3 km h^−1^ were provided; thus, a linear CoT-*v* relationship on this participant was evaluated with three running speeds. Between walking and running, the participants took a sitting rest for 7–8 min.

### Cardiorespiratory measurement and analysis

Hyperoxic air was supplied by a large custom-made Douglas bag (1000L; ARCO SYSTEM Inc., Kashiwa, Japan) through a stopcock and 1.0 m suction hose into a two-way non-rebreathing valve (Series 2700, Hans Rudolph Inc., USA). A two-way non-rebreathing valve was connected with a volume transducer that was attached to a gas collection mask. Before the hyperoxic measurement, sampling tube from the gas analyzer was inserted into the Douglas bag through a small cock to check whether the filled hyperoxic gas was 40%. Oxygen saturation of peripheral artery (SpO_2_; %) was also monitored from a right index finger during the standing rest (PULSOX-1, Konica Minolta, Japan). A stopcock was opened to inspire the room air under normoxic condition.

During walking and running, SpO_2_ and pulse rate were measured with the pulse oximeter from right index finger at the final minute of each stage. The pulse rate was regarded as the heart rate (HR; beats min^−1^). Oxygen uptake ($\dot{V}{\mathrm{O}}_2$; mL kg^−1^ min^−1^), carbon dioxide output ($\dot{V}{\mathrm{CO}}_2$; mL kg^−1^ min^−1^), ventilation (${\dot{V}}_{\mathrm{E}}$; L min^−1^), and end-tidal *P*_CO2_ (P_ET_CO_2_; mmHg) were continuously measured with a computerized breath-by-breath system (AE-310S, Minato Ltd., Japan). The FiO_2_ was measured with a paramagnetic gas analyzer with a precision of ± 0.1% O_2_. This analyzing system allowed us to evaluate end-tidal *P*_O2_ (P_ET_O_2_) from 0 to 100%. Expired carbon dioxide fraction was measured with an infrared absorption type carbon dioxide analyzer with a precision of ± 0.1% CO_2_. Well-known gas concentrations (O_2_ 15.22%, CO_2_ 5.17%, and N_2_ 79.61%) and room air were used for the calibration of the gas analyzer. Each gait speed was kept for 4 min, and a single sample of an average $\dot{V}{\mathrm{O}}_2$ and $\dot{V}{\mathrm{CO}}_2$ for the final 2 min at each gait speed was used to calculate the energy expenditure (EE; J kg^−1^ min^−1^) with the following equation [29, 30]:

1 $$ \mathrm{EE}=4.186\times \left(3.869\times \dot{V}{\mathrm{O}}_2+1.195\times \dot{V}{\mathrm{CO}}_2\right) $$

The CoT (J kg^−1^ km^−1^) values were obtained as follows:

2 $$ \mathrm{CoT}=\mathrm{EE}\times \frac{1}{60}\times \frac{1}{\mathrm{minute}\ \mathrm{speed}}\times 1000 $$

The CoT values were compared at each gait speed between normoxia and hyperoxia to evaluate whether the U-shaped and/or linear CoT-*v* relationships shifted upward or downward. A following quadratic equation was applied for a relationship between CoT and walking speeds [10, 18]:

3 $$ \mathrm{CoT}=a{v}^2+ bv+c $$

where the coefficients *a*, *b*, and *c* are determined by the least square regression with data obtained from eight walking speeds. The ES can be obtained when a differential function of the Eq. 3 (CoT’(*v*) = 2a*v* + b) is zero. Note that an alteration of the ES was used to evaluate whether the U-shaped CoT-*v* relationship shifted rightward (faster) when walking under hyperoxia (Additional file 1: Figure S1). The individual ES was determined as follows:

4 $$ \mathrm{ES}=\frac{\left|-b\right|}{2a} $$

A linear regression analysis was applied for the running CoT-*v* relationship as follows [18].

5 $$ \mathrm{CoT}= pv+q $$

where the coefficients *p* and *q* are determined by the least square regression with data from four running speeds. The EOTS is obtained as the value *v* that makes the Eqs. 3 and 5 equal. Rearranging Eqs. 3 and 5:

6 $$ {av}^2+\left(b-p\right)v+\left(c-q\right)=0 $$

Since *b* − *p* always takes negative value in Eq. 6, the absolute |*b* − *p*| is regarded as the *b* − *p*. The following equation gives two solutions of Eq. 6, and a faster one was regarded as the EOTS [18].

7 $$ \mathrm{EOTS}=\frac{-\left(b-p\right)\pm \sqrt{{\left(b-p\right)}^2-4a\left(c-q\right)}}{2a} $$

### Electromyographic measurement and analysis

After determination of the individual EOTS, electromyography (EMG) was recorded from the TA, GM, and GL during walking and running corresponding to the individual EOTS. Some previous simulation studies using motion analysis examined only the GM and *soleus*, but not GL [31]. Since ankle power generated by the *soleus* mainly functions as a vertical body support [32, 33], we investigated the GM and GL, but not the *soleus*. One of the participants on the level gradient and two of them on the uphill gradient were excluded from the analysis, because incessant noises were permeated into the EMG signal.

Pre-amplified active surface EMG electrode (BA-U410m, Nihon Santeku Co., LTD, Japan) was placed on the target muscles. Before electrode placement, the skin was shaved and wiped with alcohol for degreasing. Electric wires were secured using surgical tape not to disturb locomotion. Each participant performed “level-walk,” “level-run,” “uphill-walk,” and “uphill-run” corresponding to the individual EOTS under normoxia. About 30 steps were sampled in each experimental condition. Number of analyzed steps and time duration in each condition were summarized in Additional file 2: Table S1. Each EMG sampling was separated with 1-min standing rest besides the treadmill. When the participants completed a series of measurements under normoxia, they started to inspire hyperoxic gas for 5–6 min during the standing rest to verify whether FiO_2_ reached 40% by monitoring the gas analyzer. Thereafter, the participants performed a series of hyperoxic EMG measurements. The measurement order of gradient (level or uphill) and gait pattern (walking or running) was randomized, that is, around 15 min were sufficient for the entire EMG measurements in one participant. There was a possibility that the electric resistance between the electrode and skin might alter during the measurements due to sweating; however, it is known that the lower leg extremities were one of the hardest skin areas to sweat [34]. Considering these procedures and previous information, effects of sweating on the EMG data could be minimal for the data interpretation.

The EMG signals were amplified with a bio-amplifier (BA 1104B, Digitex Lab Co., LTD, Japan). Sampling frequency was set at 2 kHz, and a band-pass filter (8–500 Hz) was applied for the EMG signals. A foot sensor (PS-20KASF4, Kyowa Electronic Instruments Co., LTD., Japan) was inserted into a right shoe to count the number of steps, and its signal was amplified with a signal conditioner (CDV-700A, Kyowa Electronic Instruments Co., LTD, Japan). All signals from each sensor were simultaneously recorded with software (MaP 1038 ver.7.4, Nihon Santeku Co., LTD, Japan).

The sum of the rectified EMG for a certain time duration was used in some previous studies [23–25]. Since preferred step frequency is likely to be different between walking and running at the EOTS, the sum of the rectified EMG (μV sec) was normalized by time duration (sec) and number of steps [18] using the same software at the off-line mode. This parameter (μV step^−1^) was regarded as the muscle activity. A fast Fourier transform was applied for the stored band-pass filtered EMG data to evaluate alterations of mean power frequency (MPF; Hz) when switching from walking to running at the EOTS, because MPF reflects motor unit recruitment patterns [35, 36].

### Statistics

The ES and EOTS were compared between normoxia and hyperoxia on each gradient using paired *t* test. Cardiorespiratory, CoT, and EMG values were compared with two-way repeated measures ANOVA within participants on each gradient using online software (ANOVA4 on the Web, Copyright 2002 Kiriki Kenshi, Japan). If a significant *F* value was obtained, Ryan’s post hoc test was applied to the appropriate datasets. Its statistical power has been reported to be equivalent to Tukey’s post hoc test [37], and it can be used regardless of the data distribution [37]. Data were presented as mean ± SD. The statistical significance was set less than 0.05 probability level.

## Results

### CoT, ES, and EOTS

**Fig. 1:**
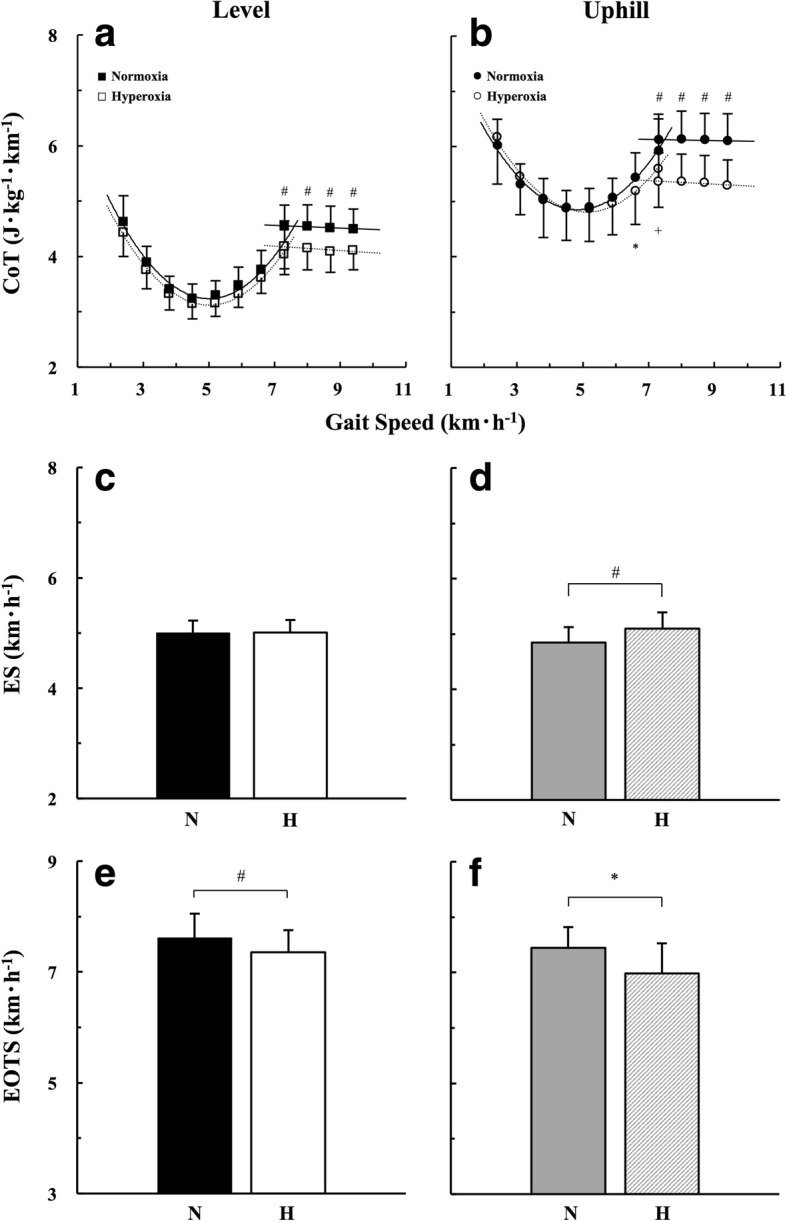
CoT-*v* relationships and comparisons of economical speed (ES) and energetically optimal transition speed (EOTS) on each gradient. **a** CoT-*v* relationships on the level and **b** uphill gradient. **c** ES on the level and **d** uphill gradient. **e** EOTS on the level and **f** uphill gradient. “N” and “H” represent normoxia and hyperoxia, respectively. ^+^*p* < 0.05, ^*^p < 0.01, and ^#^*p* < 0.001 between normoxia and hyperoxia

During walking on the uphill gradient, the CoT values were significantly lower under hyperoxia than normoxia at faster gait speeds over 6.6 km h^−1^ (Fig. 1b). During running on both gradients, the CoT values were significantly lower under hyperoxia than normoxia (Fig. 1a, b). A significantly faster ES was observed under hyperoxia (5.093 ± 0.297 km h^−1^) than normoxia (4.844 ± 0.279 km h^−1^) on the uphill gradient (Fig. 1d), while no significant difference was found between hyperoxia (5.011 ± 0.224 km h^−1^) and normoxia (5.001 ± 0.224 km h^−1^) on the level gradient (Fig. 1c). The EOTS was significantly slower under hyperoxia (7.359 ± 0.395 km h^−1^) than normoxia (7.614 ± 0.438 km h^−1^) on the level gradient (Fig. 1e), and it was also slower under hyperoxia (6.980 ± 0.547 km h^−1^) than normoxia (7.446 ± 0.374 km h^−1^) on the uphill gradient (Fig. 1f).

### Cardiorespiratory response

**Fig. 2:**
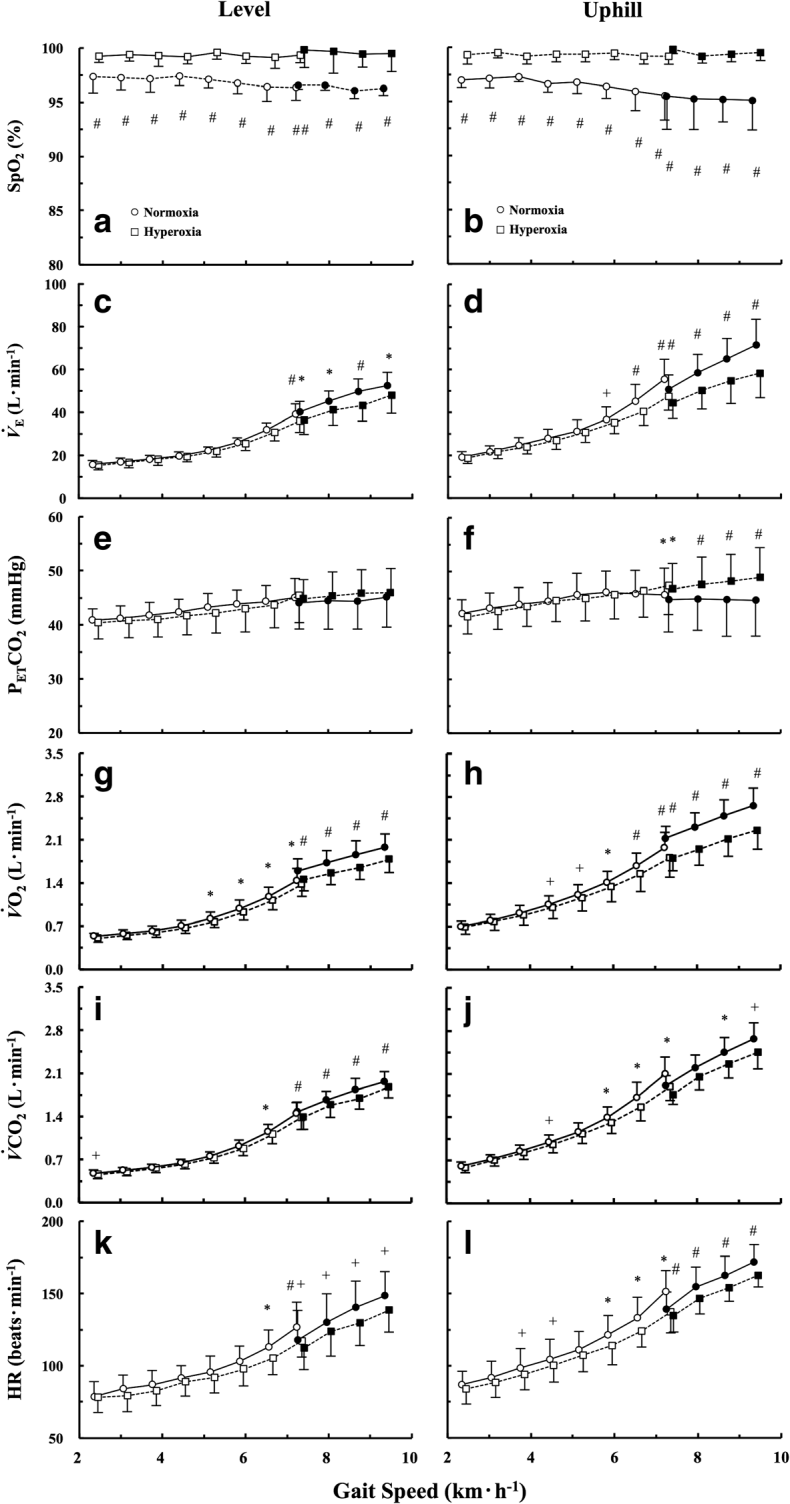
Comparisons of cardiorespiratory parameters between normoxia and hyperoxia on each gradient. **a** SpO_2_ on the level and **b** uphill gradient. **c** *V*_E_ on the level and **d** uphill gradient. **e** P_ET_CO_2_ on the level and **f** uphill gradient. **g** $\dot{V}{\mathrm{O}}_2$ on the level and **h** uphill gradient. **i** $\dot{V}{\mathrm{CO}}_2$ on the level and **j** uphill gradient. **k** HR on the level and **l** uphill gradient, respectively. Open circles and squares represent walking under normoxia and hyperoxia. Filled circles and squares represent running under normoxia and hyperoxia. To avoid overlapping plots at each speed, normoxic and hyperoxic data were plotted 0.1 km h^−1^ slower or faster than the actual speeds. ^+^*p* < 0.05, ^*^p < 0.01, and ^#^*p* < 0.001 between normoxia and hyperoxia

Cardiorespiratory results were presented in Fig. 2. In brief, significantly higher oxygen saturation of peripheral artery (SpO_2_; %) was observed under hyperoxia than normoxia at any gait speeds on both gradients (Fig. 2a, b). *V*_E_ was significantly lower under hyperoxia than normoxia over 7.3 km h^−1^ on the level gradient and over 5.9 km h^−1^ on the uphill gradient (Fig. 2c, d). P_ET_CO_2_ was significantly higher under hyperoxia than normoxia during walking at 7.3 km h^−1^ and all running speeds on the uphill gradient (Fig. 2f). $\dot{V}{\mathrm{O}}_2$ was significantly lower under hyperoxia over 5.2 km h^−1^ on the level gradient (Fig. 2g) and over 4.5 km h^−1^ on the uphill gradient (Fig. 2h). $\dot{V}{\mathrm{CO}}_2$ was significantly lower under hyperoxia at several gait speeds (Fig. 2i, j). HR was significantly lower under hyperoxia than normoxia on both gradients mainly at faster gait speeds (Fig. 2k, l).

### Muscle activity and MPF at the EOTS

**Fig. 3:**
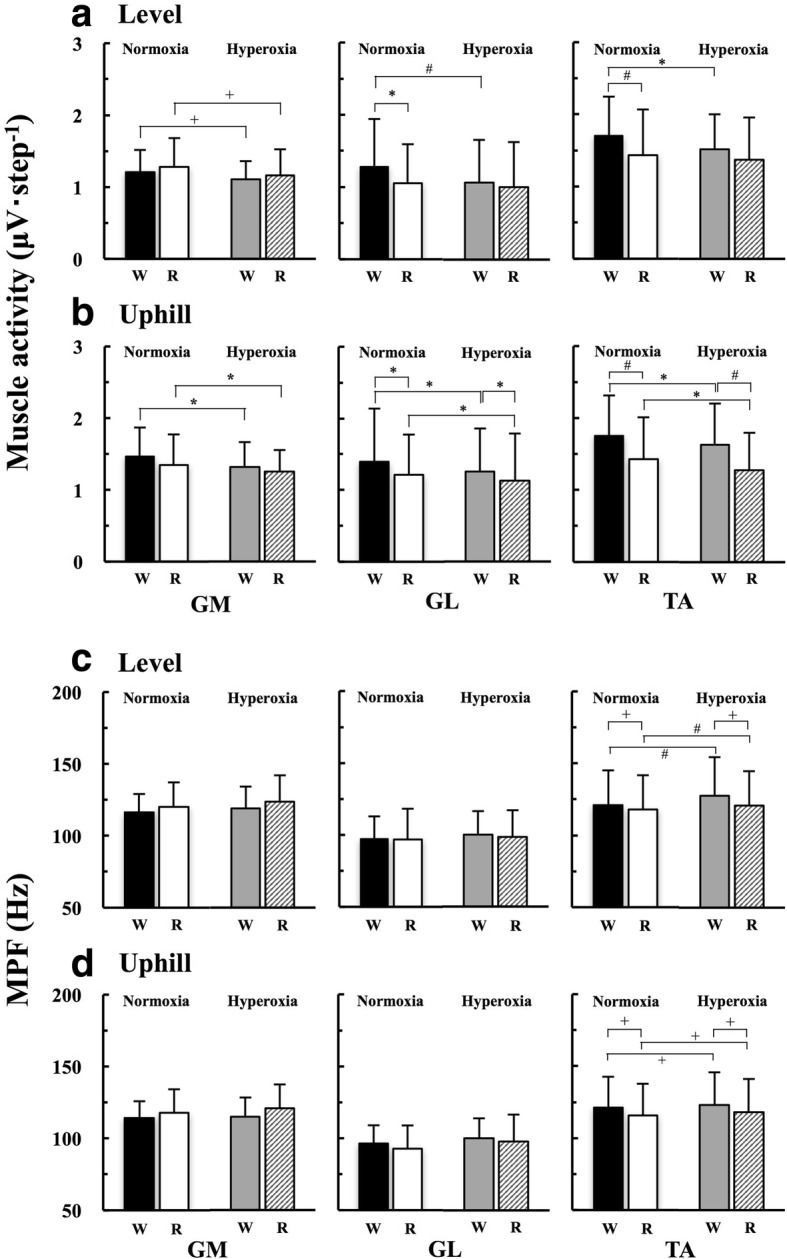
Comparisons of muscle activities and mean power frequency (MPF) at the energetically optimal transition speed (EOTS) on each gradient. **a** Muscle activities on the level and **b** uphill gradient. **c** MPF on the level and **d** uphill gradient. “W” and “R” represent walking and running, respectively. ^+^*p* < 0.05, ^*^p < 0.01, and ^#^*p* < 0.001

TA activity became significantly lower when switching from walking to running at the EOTS on the level gradient under normoxia and uphill gradient under both FiO_2_ conditions (Fig. 3a, b). GM activity was not significantly different between walking and running on both gradients (Fig. 3a, b). GL activity became significantly lower during running than walking at the EOTS on both gradients under hyperoxia (Fig. 3a, b), but not on the level gradient under hyperoxia. MPF of the GM and GL was not significantly different between walking and running, while MPF of the TA was lower during running than walking on both gradients (Fig. 3c, d). TA, GM, and GL activities under normoxia were significantly lower than those under hyperoxia (Fig. 3a, b) except GL and TA activities during running on the level gradient. MPF of the TA was significantly higher under normoxia than hyperoxia during both gaits on both gradients (Fig. 3c, d), although no significant difference was observed in the GL and GM.

## Discussion

### Economical speed

A higher SpO_2_ under hyperoxia than normoxia suggested that our experimental setup successfully established a hyperoxic condition (Fig. 2a, b). In support of the former part of our first hypothesis as shown in Additional file 1: Figure S1, the ES was significantly faster under hyperoxia than normoxia on the uphill gradient (Fig. 1b, d), but not on the level gradient (Fig. 1a, c).

An existence of the ES is one of the few biological advantages of the erect bipedalism, because healthy walkers naturally selected their ES as a preferred walking speed [4, 38]. Figueiredo et al. [39] suggested a significant contribution of increased ventilatory responses for self-selected walking speed, which could be close to the ES. In our present study, hyperoxia was supposed to depress the peripheral chemoreceptor activation (i.e., chemoreflex drive), which could reduce ventilatory and HR responses as shown in Fig. 2c, d, k, and l. It should be noted that hyperoxia also reduces adenosine triphosphate (ATP) synthesis rate and concomitant reduction in mitochondrial efficiency during dynamic exercise at moderate intensity [40]. Thus, there is a possibility that hyperoxia directly works with the reduced metabolic demand during human locomotion.

Since the CoT values were mainly determined by $\dot{V}{\mathrm{O}}_2$ as shown in Eq. 1 [29, 30], the lower CoT values during walking under hyperoxia were mainly attributed to a reduced $\dot{V}{\mathrm{O}}_2$ rather than $\dot{V}{\mathrm{CO}}_2$ (Fig. 2i, j). These consequences were particularly prominent at faster walking speeds on the uphill gradient, because they explain the rightward (faster) shift of the overall shape of the U-shaped CoT-*v* curve under hyperoxia (Fig. 1b). As the shape of the U-shaped CoT-*v* curve determined the ES as shown in Additional file 1: Figure S1, its overall shape in walking remained unchanged on the level gradient, resulting in an unchanged ES on that gradient (Fig. 1a, c).

### Energetically optimal transition speed

Current anthropological literatures on the EOTS have been focused whether the human beings change their gait pattern only for a minimization of the whole-body energy expenditure [6, 11, 13, 19–22]. To our surprise, the EOTS was significantly slower under hyperoxia than normoxia (Fig. 1e, f), suggesting that the latter part of our first hypothesis was rejected. The results suggest that running was more economical than walking under hyperoxia. In fact, around 10% lower CoT values were observed during running under hyperoxia than normoxia (Fig. 1a, b; ranged from 9.1 to 10.7% on the level gradient and from 12.2 to 13.2% on the uphill gradient, respectively). These relatively lower CoT values during running caused a drastic downward shift of the linear CoT-*v* relationship in running, resulting in a significantly slower EOTS under hyperoxia on both gradients (Fig. 1e, f).

A preferred gait transition occurred around 7–8 km h^−1^ on the level gradient under normoxia in adult males [11, 12, 19–22]. Our recent study observed that the EOTS was equivalent between moderate hypoxia (15% FiO_2_) and normoxia on any gradients [18]. However, as far as we know, there has currently been no comparable data of the EOTS under hyperoxia. It should be noted that the preferred gait transition speed was around 5–6% slower than the EOTS [11, 19–21], suggesting that “energetically optimal” may not be a necessary and sufficient condition for the human gait transition.

### Comparison of muscle activity between walking and running

In support of our second hypothesis, TA activity significantly decreased when the gait pattern was switched from walking to running at the EOTS under normoxia on both gradients (Fig. 3a, b), but not under hyperoxia on the level gradient (Fig. 3a). These results, which were observed under normoxia, were consistent with the results of some previous studies [18, 23–25]. It is worth noting that the MPF of the TA was lower during running than walking (Fig. 3c, d), suggesting that the motor unit recruitment pattern of the TA shifted toward more type I (slow twitch) fibers rather than type II (fast twitch) fibers when switching the gait pattern from walking to running at the EOTS, because motor unit recruitment patterns are reflected by MPF [35, 36].

Some considerations are necessary, because the TA mainly activates during swing phase. Instead of the TA, plantar flexors (GM and GL) play a substantial role in forward acceleration during walking [27, 33, 41, 42] and running [32]. Reduced plantar flexor activity during the push-off phase resulted in a decrease in the CoT [43, 44]. These previous findings indicated that plantar flexor activity was mainly responsible for the CoT during walking. Note that the GM, an antagonist of the TA, has been proposed to play a key role in triggering the gait transition, because the force production by the GM decreased as a function of walking speed [31, 41]. As shown in Fig. 3a, b, GM activity was not significantly different between walking and running on either gradient irrespective of FiO_2_ conditions. It was surprising to observe that GL activity significantly decreased when the gait pattern was switched from walking to running except on the level gradient under hyperoxia (Fig. 3a, b), although reduced GL activity with unchanged GM activity could be associated with a possible increase in the muscle contraction velocity [41]. Indeed, time duration of one gait cycle was nearly 14% shorter during running than walking under all conditions (Additional file 2: Table S1). In addition, the MPF of both GM and GL was not significantly different between walking and running at the EOTS (Fig. 3c, d). These results demonstrated that the motor unit recruitment patterns in these synergists remained unchanged, being supported by the results of our recent study [18].

The GM and GL may share the necessary muscle activations without alterations of motor unit recruitment pattern. Planter flexors are divided into three synergetic muscles (GM, GL, and *soleus*), so that the total mass of these synergists seems to be biologically redundant if compared to other bipedal species [14, 15]. Such a possible biological redundancy of these planter flexors can contribute to avoid early onset of localized muscle fatigue. In contrast, the TA should have a high endurance capacity, as more than 70% of the TA consisted of slow twitch fibers only in the humans [45, 46]. This histochemical feature of the TA must be one of the evolutionary adaptations caused by the erect bipedalism. Once the TA recruited all type I (slow and endurance) fibers, it is necessary to recruit type II (fast twitch) fibers. Thus, TA activity and its motor unit recruitment pattern are likely to be more influenced by gait patterns compared to the plantar flexors. Such interpretations do not conflict with a theory of Henneman’s size principle [47]. Considering above, complicated lower leg muscle activities and those motor unit recruitment patterns observed in our study suggested that not only TA but also GL may be related to the gait selection either walking or running around the EOTS.

### Comparison of muscle activity between normoxia and hyperoxia

Our present study observed that the muscle activities were lower under hyperoxia than normoxia regardless of gait patterns (Fig. 3a, b), suggesting that our second hypothesis was partly rejected. Since EMG measured from lower leg extremities during human locomotion is highly sensitive to the gait speed [23], these reduced muscle activities should be mainly attributed to 3.35% (level gradient; Fig. 1e) and 6.26% (uphill gradient; Fig. 1f) slower EOTS under hyperoxia. It is a noteworthy fact that muscular activities at a given work rate tended to decrease under hyperoxia due to a reduced ATP synthesis rate in the exercising muscles [40]. Considering these facts, a slower EOTS in association with lower muscle activities caused by hyperoxia could be partly accounted for the lower energy expenditure under hyperoxia than normoxia.

It is more interesting to note that the MPF was still higher under hyperoxia than normoxia only in the TA (Fig. 3c, d), although the EOTS was slower under hyperoxia than normoxia on both gradients (Fig. 1e, f). These results demonstrated that more fast-twitch fibers were recruited under hyperoxia than normoxia only in the TA. Here, our primary question is why only TA is sensitive to gait patterns and FiO_2_ conditions. First, the centroidal line goes through the TA when standing [48]. Second, relative muscle weight of the TA is less than 20% of the plantar flexors in humans [49]. These anatomical characteristics of the TA will be one aspect for explaining greater sensitivity of the TA during human locomotion.

Another aspect should be also considered. Very few potential studies are partly related to our study [50, 51]. Amann et al. [50] measured EMG from three thigh muscles during maximal cycle ergometer exercise under 100% FiO_2_ condition. Peltonen et al. [51] also measured EMG from several leg muscles during maximal rowing ergometer exercise under 62.2% FiO_2_ condition. It is necessary to take into account that the measured muscles, exercise mode and/or intensity, FiO_2_ levels, and experimental protocols in these previous studies [50, 51] were different from the present study. Note that both previous studies observed significantly higher MPF values in the main working muscles under hyperoxia [50, 51], suggesting that type II fibers are likely to be recruited under hyperoxia. In fact, hyperoxia activates group IV muscle afferents [52].

As mentioned before, the TA mainly activates in the swing phase [23–26]; however, to the best of our knowledge, its motor unit recruitment characteristics during human locomotion under different FiO_2_ conditions have not been yet well investigated except for our recent study [18]. Supplementary oxygen after muscle fatigue was beneficial for sustained force production [28]. Moderate hyperoxic gas inspiration can avoid or soften acute mountain sickness while mountaineering [53]. Therapeutic hyperoxia has been reported to be available for patients with chronic obstructive pulmonary disease [54]. It is interesting to note that the cardiorespiratory and/or muscular responses during exercise under hyperoxia exhibited large individual differences [28, 50, 54]. These results suggest that more investigations are necessary for muscle activities and cardiorespiratory responses during human locomotion under hyperoxia.

### Limitations

Our treadmill cannot control gait speeds in a ramp manner, so that a natural gait transition speed could not be observed in this study. This limitation further resulted in another limitation whether the EOTS corresponded to the actual gait transition speed.

## Conclusions

Hyperoxia decreased CoT values particularly at faster walking speeds and any running speeds, being responsible for the faster ES on the uphill gradient and slower EOTS on both gradients. Reduced GL and TA activities in association with a lower shift of the motor unit recruitment pattern in the TA would be related to the gait selection when walking or running at the EOTS.

## Additional files

Additional file 1: **Figure S1.** Schematic illustration of cost of transport (CoT) and gait speed (*v*) under normoxia and hyperoxia. Combination of downward and rightward shifts of the U-shaped CoT-gait speed (*v*) relationship under hyperoxia is presented. Arrows mean potential shifting directions. (JPG 737 kb)

Additional file 2: **Table S1.** Summary of analyzed steps, its time duration, step frequency, and gait cycle. Values are mean ± SD. Step frequency was significantly higher during running than walking at any conditions. (JPG 484 kb)

## Abbreviations

ANOVA: Analysis of variance
CoT: Cost of transport
EE: Energy expenditure
EMG: Electromyography
EOTS: Energetically optimal transition speed
ES: Economical speed
FiO_2_: Fraction of inspired oxygen
GL: Gastrocnemius lateralis
GM: Gastrocnemius medialis
HR: Heart rate
MPF: Mean power frequency
P_ET_CO_2_: End-tidal pressure of carbon dioxide
SpO_2_: Oxygen saturation of peripheral artery
TA: Tibialis anterior
$\dot{V}{\mathrm{CO}}_2$: Carbon dioxide output
$$ {\dot{V}}_{\mathrm{E}} $$: Minute ventilation
$$ \dot{V}{\mathrm{O}}_2 $$: Oxygen uptake

## References

[1] Sockol MD, Raichlen DA, Pontzer H (2007). Chimpanzee locomotor energetics and the origin of human bipedalism. PNAS.

[2] Bramble DM, Lieberman DE (2004). Endurance running and the evolution of Homo. Nature.

[3] Abe D, Fukuoka Y, Horiuchi M (2015). Economical speed and energetically optimal transition speed evaluated by gross and net oxygen cost of transport at different gradients. PLoS One.

[4] Wezenberg D, van der Woude LH, Faber WX, de Haan A, Houdijk H (2013). Relation between aerobic capacity and walking ability in older adults with a lower-limb amputation. Arch Phys Med Rehabil.

[5] Cavagna GA, Saibene FP, Margaria R (1963). External work in walking. J Appl Physiol.

[6] Minetti AE, Capelli C, Zamparo P, di Prampero PE, Saibene F (1995). Effects of stride frequency on mechanical power and energy expenditure of walking. Med Sci Sports Exerc.

[7] Abe D, Muraki S, Yasukouchi A (2008). Ergonomic effects of load carriage on the upper and lower back on metabolic energy cost of walking. Appl Ergon.

[8] Horiuchi M, Endo J, Horiuchi Y, Abe D (2015). Comparisons of energy cost and economical walking speed at various gradients in healthy, active younger and older adults. J Exerc Sci Fit.

[9] Griffin TM, Tolani NA, Kram R (1999). Walking in simulated reduced gravity: mechanical energy fluctuations and exchange. J Appl Physiol.

[10] Horiuchi M, Handa Y, Abe D, Fukuoka Y (2016). Walking economy at simulated high altitude in human healthy young male lowlanders. Biol Open.

[11] Hreljac A (2013). Preferred and energetically optimal gait transition speeds in human locomotion. Med Sci Sports Exerc.

[12] Minetti AE, Ardigò LP, Saibene F (1994). The transition between walking and running in humans: metabolic and mechanical aspects at different gradients. Acta Physiol Scand.

[13] Usherwood JR, Bertram JE (2003). Gait transition cost in humans. Eur J Appl Physiol.

[14] Watson RR, Rubenson J, Coder L, Hoyt DF, Propert MW, Marsh RL (2011). Gait-specific energetics contributes to economical walking and running in emus and ostriches. Proc Biol Sci.

[15] Daley MA, Channon AJ, Nolan GS, Hall J (2016). Preferred gait and walk-run transition speeds in ostriches measured using GPS-IMU sensors. J Exp Biol.

[16] Scheuermann BW, Kowalchuk JM, Paterson DM, Cunningham DA (1999). Peripheral chemoreceptor function after carbonic anhydrase inhibition during moderate-intensity exercise. J Appl Physiol.

[17] Segizbaeva MO, Aleksandrova NP (2009). Effects of oxygen breathing on inspiratory muscle fatigue during resistive load in cycling men. J Physiol Pharmacol.

[18] Abe D, Fukuoka Y, Horiuchi M (2017). Muscle activities during walking and running at energetically optimal transition speed under normobaric hypoxia on gradient slopes. PLoS One.

[19] Tseh W, Bennett J, Caputo JL, Morgan DW (2002). Comparison between preferred and energetically optimal transition speeds in adolescents. Eur J Appl Physiol.

[20] Rotstein A, Inbar O, Berginsky T, Meckel Y (2005). Preferred transition speed between walking and running: effects of training status. Med Sci Sports Exerc.

[21] Ganley KJ, Stock A, Herman RM, Santello M, Willis WT (2011). Fuel oxidation at the walk-to-run transition in humans. Metabolism.

[22] Ziv G, Rotstein A (2009). Physiological characteristics of the preferred transition speed in racewalkers. Med Sci Sports Exerc.

[23] Prilutsky BI, Gregor RJ (2001). Swing- and support-related muscle actions differentially trigger human walk-run and run-walk transitions. J Exp Biol.

[24] Bartlett JL, Kram R (2008). Changing the demand on specific muscle groups affects the walk-run transition speed. J Exp Biol.

[25] Malcolm P, Segers V, Van Caekenberghe I, De Clercq D (2009). Experimental study on the influence of the m. tibialis anterior on the walk-to-run transition by means of powered ankle-foot exoskeleton. Gait Posture.

[26] Segers V, De Smet K, Van Caekenberghe I, Aerts P, De Clercq D (2013). Biomechanics of spontaneous overground walk-to-run transition. J Exp Biol.

[27] Gottschall JS, Kram R (2003). Energy cost and muscular activity required for propulsion during walking. J Appl Physiol.

[28] Shimoda M, Enomoto M, Horie M, Miyakawa S, Yagishita K (2015). Effects of hyperbaric oxygen on muscle fatigue after maximal intermittent plantar flexion exercise. J Strength Cond Res.

[29] Brouwer E (1957). On simple formulae for calculating the heat expenditure and the quantities of carbohydrate and fat oxidized in metabolism of men and animals, from gaseous exchange (Oxygen intake and carbonic acid output) and urine-N. Acta Physiol Pharmacol Neerl.

[30] Masschelein E, Van Thienen R, Wang X, Van Schepdael A, Thomis M, Hespel P (2012). Dietary nitrate improves muscle but not cerebral oxygenation status during exercise in hypoxia. J Appl Physiol.

[31] Neptune RR, Sasaki K (2005). Ankle plantar flexor force production is an important determinant of the preferred walk-to-run transition speed. J Exp Biol.

[32] Hamner SR, Seth A, Delp SL (2010). Muscle contributions to propulsion and support during running. J Biomech.

[33] Franz JR, Thelen DG (2016). Imaging and simulation of Achilles tendon dynamics: implications for walking performance in the elderly. J Biomech.

[34] Smith CJ, Havenith G (2011). Body mapping of sweating patterns in male athletes in mild exercise-induced hyperthermia. Eur J Appl Physiol.

[35] De Luca CJ (1997). The use of surface electromyography in biomechanics. J Appl Biomech.

[36] Wakeling JM (2009). Patterns of motor unit recruitment can be determined using surface EMG. J Electromyogr Kinesiol.

[37] Ryan TA (1960). Significance tests for multiple comparison of proportions, variances, and other statistics. Psychol Bull.

[38] Wall-Scheffler CM, Myers MJ (2013). Reproductive costs for everyone: how female loads impact human mobility strategies. J Hum Evol.

[39] Figueiredo P, Ribeiro PA, Bona RL, Peyré-Tartaruga LA, Ribeiro JP (2013). Ventilatory determinants of self-selected walking speed in chronic heart failure. Med Sci Sports Exerc.

[40] Layec G, Trinity JD, Hart CR, Kim SE, Groot HJ, Le Fur Y, Sorensen JR, Jeong EK, Richardson RS (2015). Impact of age on exercise-induced ATP supply during supramaximal plantar flexion in humans. Am J Physiol.

[41] Farris DJ, Sawicki GS (2012). Human medial gastrocnemius force-velocity behavior shifts with locomotion speed and gait. PNAS.

[42] Francis CA, Lenz AL, Lenhart RL, Thelen DG (2013). The modulation of forward propulsion, vertical support, and center of pressure by the plantarflexors during human walking. Gait Posture.

[43] Collins SH, Wiggin MB, Sawicki GS (2015). Reducing the energy cost of human walking using an unpowered exoskeleton. Nature.

[44] Huang TW, Shorter KA, Adamczyk PG, Kuo AD (2015). Mechanical and energetic consequences of reduced ankle plantar-flexion in human walking. J Exp Biol.

[45] Dahmane R, Djordjevic S, Simunic B, Valencic V (2005). Spatial fiber type distribution in normal human muscle: histochemical and tensiomyographical evaluation. J Biomech.

[46] Johnson MA, Polgar J, Weightman D, Appleton D (1973). Data on the distribution of fibre types in thirty-six human muscles. J Neurol Sci.

[47] Henneman E, Somjen G, Carpenter DO (1965). Functional significance of cell size in spinal motoneurons. J Neurophysiol.

[48] Kimura T (1996). Centre of gravity of the body during the ontogeny of chimpanzee bipedal walking. Folia Primatol.

[49] Ito J (1996). Morphological analysis of the human lower extremity based on the relative muscle weight. Okajimas Folia Anat Jpn.

[50] Amann M, Romer LM, Pegelow DF, Jacques AJ, Hess CJ, Dempsey JA (2006). Effects of arterial oxygen content on peripheral locomotor muscle fatigue. J Appl Physiol.

[51] Peltonen JE, Rusko HK, Rantamäki J, Sweins K, Niittymäki S, Viitasalo JT (1997). Effects of oxygen fraction in inspired air on force production and electromyogram activity during ergometer rowing. Eur J Appl Physiol.

[52] Arbogast S, Vassilakopoulos T, Darques JL, Duvauchelle JB, Jammes Y (2000). Influence of oxygen supply on activation of group IV muscle afferents after low-frequency muscle stimulation. Muscle Nerve.

[53] Davis C, Hackett P (2017). Advances in the prevention and treatment of high altitude illness. Emerg Med Clin North Am.

[54] Queiroga F, Nunes M, Meda E, Chiappa G, Machado MC, Nery LE, Neder JA (2013). Exercise tolerance with helium-hyperoxia versus hyperoxia in hypoxaemic patients with COPD. Eur Respir J.

