# Supplementary figures and images for: Energy cost and lower leg muscle activities during erect bipedal locomotion under hyperoxia
Source: J Physiol Anthropol. 2018 Jun 19;37:18. doi: 10.1186/s40101-018-0177-7 (PMC6006575; doi:10.1186/s40101-018-0177-7)

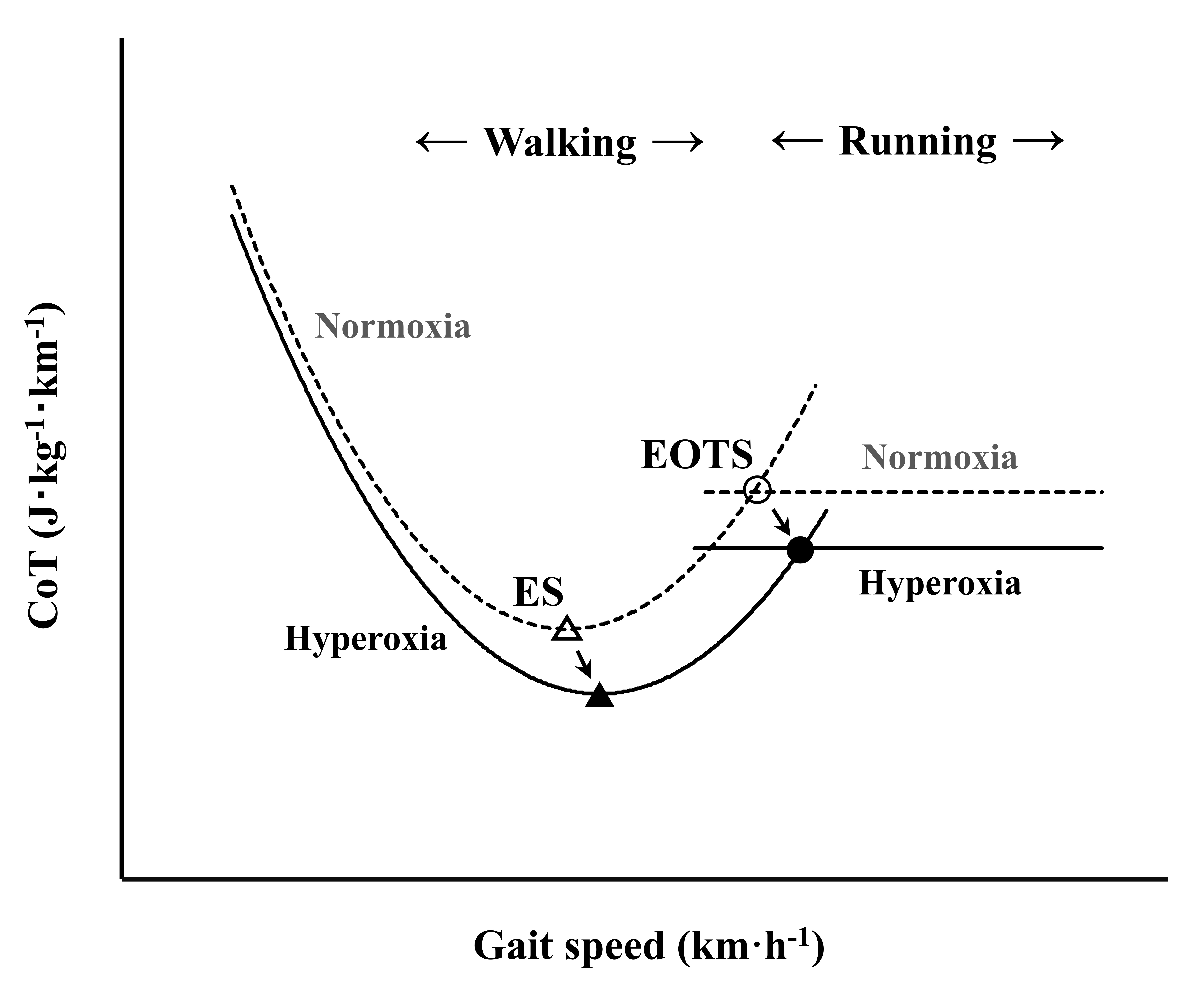

Supplement: Supplementary file 1 — Figure S1. Schematic illustration of cost of transport (CoT) and gait speed (v) under normoxia and hyperoxia. Combination of downward and rightward shifts of the U-shaped CoT-gait speed (v) relationship under hyperoxia is presented. Arrows mean potential shifting directions. (JPG 737 kb) [file 40101_2018_177_MOESM1_ESM.jpg]

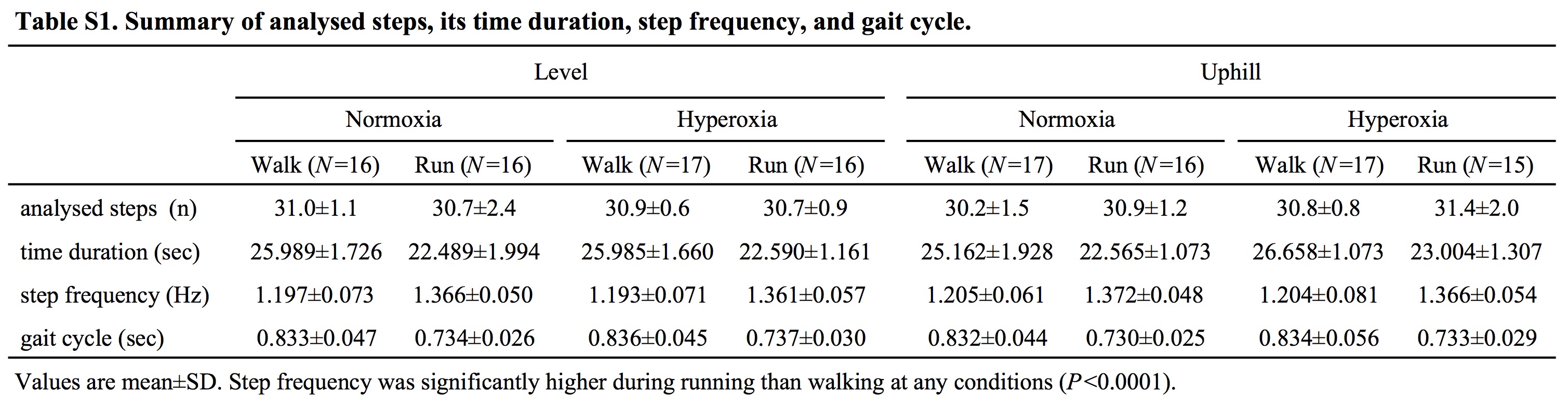

Supplement: Supplementary file 2 — Table S1. Summary of analyzed steps, its time duration, step frequency, and gait cycle. Values are mean ± SD. Step frequency was significantly higher during running than walking at any conditions. (JPG 484 kb) [file 40101_2018_177_MOESM2_ESM.jpg]
